# Antigenicity evaluation of lac color and exploratory study for identifying potential biomarkers of anaphylaxis

**DOI:** 10.1186/s42826-024-00229-z

**Published:** 2024-11-26

**Authors:** Hyun-Jin Lim, Kang Min Han, Seung-Hyun Kim, Soo-Kyung Ryu, Ji-Ran You, Jung-Hee Yoon, Euna Kwon, Ji-Eun Kim, Byeong-Cheol Kang

**Affiliations:** 1 Laboratory Animal Medicine, Graduate School of Translational Medicine, College of Medicine, Seoul National University, 103, Daehak-ro Jongno-gu, Seoul, Republic of Korea; 2 Department of Experimental Animal Research, Biomedical Research Institute, Seoul National University Hospital, Seoul, Republic of Korea; 3 Department of Pathology, CHA Ilsan Medical Center, Goyang-si, Republic of Korea

**Keywords:** Exploratory study, Lac color, Antigenicity, Active systemic anaphylaxis, Biomarkers

## Abstract

**Background:**

Lac color, a natural red dye derived from the larvae of laccifer lacca kerr, is one of the most commonly used substances in food. To date, no studies have reported on the antigenicity of lac color and the other biomarkers that can determine anaphylactic reactions. To address this, we evaluated the antigenicity of lac color through active systemic anaphylaxis (ASA) in addition to identifying potential biomarkers performing exploratory studies. For ASA test, Guinea pigs (n = 5) were sensitized with 0(negative control), 4 mg/kg of lac color, 4 mg/kg of lac color + FCA, and 5 mg/kg of ovalbumin + FCA (positive control) 3 times a week for three weeks. Fourteen days after the last sensitization, animals were challenged intravenously weekly for two weeks. Hematological and histopathological analyses were performed and compared to control groups.

**Results:**

In the ASA test, all lac color groups showed mild symptoms such as nose rubbing, urination, and evacuation, which are insufficient indicators of anaphylaxis. Exploratory studies identified several biomarkers: decreased platelet count, and increased basophil count; distention in the lung, and redness on the inner wall of trachea; mononuclear inflammatory cell infiltration (MICI) in the ear, and heart hemorrhage. When these biomarkers were applied to the ASA test of lac color, in comparison to the negative control group, the positive control group (ovalbumin + FCA) showed a significant over 60-fold reduction in platelet count and nearly threefold higher basophil count compared to other groups. Furthermore, only positive control group exhibited full lung distention and severe redness on the inner wall of the trachea. Mononuclear inflammatory cell infiltration (MICI) in the ear was about three times higher, and heart hemorrhage was only present in the positive control group compared to others. None of the lac color groups were different from the negative control group (*p* > 0.05), whereas the positive control group was significantly different (*p* < 0.05).

**Conclusions:**

Our study concludes that lac color, at the tested concentrations, does not induce antigenicity in the guinea pig model, providing valuable safety data. Furthermore, the biomarkers identified in this study offer a supportive approach to evaluating the immunogenicity of substances in future research.

**Supplementary Information:**

The online version contains supplementary material available at 10.1186/s42826-024-00229-z.

## Background

Food colorings, also known as edible colors, are any dye or pigment that imparts color to food or drink to improve its appearance [1]. The history of food coloring dates back to 1500 BC in ancient Egypt, where colored candies were depicted in paintings found in tombs [2]. In modern times a variety of food colorings have been developed which are largely classified as either artificial or natural. Natural colors are defined as pigments, dyes, or any other substances that originate from natural sources [3]. It is believed that natural colors were extensively used before artificial colors emerged, as they are sourced from readily available materials like animals and plants in nature. These food colorings are added to millions of products such as ice cream, soft drink, milk, sausages, fish, baked goods, fruits, and so forth [3]. This means that food colorings are extremely prevalent in our daily lives, and consumers’ concerns about the safety of artificial colors have given impetus to the development of alternative colors [1, 4].

However, non-negligible problems with natural colors have begun to be pointed out. Among the red-series colors, carmine, also called cochineal extract, has been reported to cause allergic reactions. Carmine is derived from the dried female bodies of the insect *Dactylopius coccusvar*. *Costa*, and consists of a core anthraquinone structure which is used in dyeing applications by the pharmaceutic, cosmetic and food industries [5, 6]. The allergenic properties of carmine were first reported by Quirce et al. in 1994, and subsequent studies by Burge and Dietemann-Molard associated carmine with occupational asthma [7, 8] and extrinsic allergic alveolitis [9]. More alarmingly, cases of anaphylaxis triggered by carmine have been documented by Wüthrich et al. in 1997 [10] (Table 1). According to food & food additive production records by Ministry of food and drug safety (MFDS), the domestic sales volume of carmine has diminished almost by half (48.9%) in 2018 compared to 2011 [11, 12] (Supplementary Fig. 1A). This decline is attributed to safety concerns associated with carmine. In contrast, the use of lac color, a red-series color similar to carmine, has significantly increased by about two-fold (209.9%) during the same period, as indicated by MFDS data. [11, 12] (Supplementary Fig. 1A).

**Table 1:** Health risks of food colorings

Category	Coloring	Molecular base	Health Risks	References
Artificial	Tartrazine	Azo	Rash, asthma, vasculitis, and contact dermatitis	[47]
	Brilliant black	Dizao	Hypersensitivity reactions, genotoxic properties	[48]
	Fast green	Triarylmethane	Skin irritation, eye irritation, and respiratory tract and digestive irritation	[48]
	Nitrates	Nitrogen, Oxygen	Carcinogenic	[49]
Natural	Carmine	Anthraquinone	Occupational asthma	[7, 8]
			Extrinsic allergic alveolitis	[9]
			Anaphylaxis	[10]

Lac color, also called Natural Red 25, is an ancient red dye with historical use dating back to the third century B.C., as found on an Italian wine jug [13]. Lac color is derived from the resinous secretions of *laccifer lacca kerr* larvae. Its primary component is Laccaic acid (C26H19NO12), which consists of five anthraquinone carboxylic acids (A, B, C, D, and E) with similar structures and the main components are known as A and B (Supplementary Fig. 1B and 1C) [14]. These days, lac color is used widely in cosmetics, drugs, textiles and foods. It is used in foods for red-colored items such as ham, sausage, jam, soft drinks, and other everyday ingredients in our meals [15]. At present, the acceptable daily intake (ADI) was established at 2 mg/kg/day in India but daily intakes have not been established by the European Food Safety Authority (EFSA), the Joint FAO/WHO Expert Committee on Food Additives (JECFA), or MFDS in Korea.

Despite its widespread use, lac color’s safety remains a concern and limited safety studies on lac color have been conducted. Dube et al. suggested that laccaic acid, found in lac color, may act as a tumor promoter, disrupting metabolic cooperation in Chinese Hamster V79 cells at 10 mg/ml [16] and others confirmed a clastogenic effect in mice bone marrow cells treated with lac color [17] in addition to an increase in the absolute and relative weights of the salivary glands in a chronic toxicity study in rats [18]. However, to date, the antigenicity of lac color has not been studied, and given that it shares certain properties with carmine such as being insect-derived and having an anthraquinone structure, should be evaluated for antigenicity.

Antigenicity is the property of being capable of binding specifically to immune components, and there are two types of immune phenomena related to antigenicity, one being allergenicity and the other, immunogenicity. Immunogenicity refers to the ability to trigger a protective immune response and allergenicity is defined as the ability of an antigen to trigger hypersensitivity reactions [19, 20]. Anaphylaxis is a severe and rapid systemic hypersensitivity reaction characterized by life-threatening symptoms such as breathing difficulties, vasodilation, increased mucus secretion, and skin changes [21]. According to MFDS, both the active systemic anaphylaxis (ASA) test and the passive cutaneous anaphylaxis (PCA) test are employed to assess the antigenicity of test substances [22]. PCA tests the local anaphylaxis by ‘passively’ sensitizing with an antiserum from other sensitized animals.

*Lac* has been reported to lack antigenicity in the PCA test [23]. However, since the PCA test does not assess the anaphylactic reaction caused by ‘actively’ produced IgE antibodies after systemic sensitization of the entire body, it is necessary to evaluate the anaphylactic potential using the ASA test. ASA uses behavioral indicators to identify systemic symptoms, but these indicators are problematic due to their subjective nature, difficulty in quantification, and vagueness in distinguishing symptoms from naturally occurring behavior such as rubbing or licking the nose, urination, and evacuation. However, these activities are considered inherent and normal in the behavioral repertoire of guinea pigs. In the studies by Park et al., and Choi et al., urination and/or evacuation in addition to the rubbing or licking nose were also observed in a significant proportion of animals in the control group [24, 25]. Thus, these symptoms are considered doubtful as positive indicators. Therefore, it is proposed that these symptoms need to be judged comprehensively in conjunction with other reliable analyses for a more accurate assessment of anaphylaxis. In this study, we employed the active systemic anaphylaxis (ASA) test in guinea pigs and complemented our analysis with additional hematological and histological markers. These markers were chosen based on their correlation with both positive and negative responses to anaphylactic materials.

## Methods

### Test substance

Lac color was purchased from Hefei Light Industrial Products Arts & Crafts I/E Co. Ltd (Hefei, China) considering that the company accounts for the largest share among companies supplying lac color in Korea, producing seven out of the 29 types distributed. Dimethyl sulfoxide (DMSO) was purchased from Sigma-Aldrich (Germany) to dissolve lac color and ovalbumin. Ovalbumin (OVA) was purchased from Sigma-Aldrich (Germany) for administration of positive control group. Freund’s complete adjuvant (FCA), used as an adjuvant to enhance the immune response, was purchased from Sigma-Aldrich (Germany) and contains 1 mg of heat-killed Mycobacterium tuberculosis in 1 mL of solution. The test substances were freshly prepared on the day of administration as follows: For oral administration of the negative control group, 0.9% normal saline was prepared. For oral administration of lac color group, lac color was dissolved with 0.9% normal saline adding glass beads for even suspension. For subcutaneous administration of lac color + FCA group, lac color was first prepared at two-fold the target dose with 0.9% normal saline. Then, shortly before administration, the stock was emulsified with the same volume of FCA, resulting in a final concentration of 4 mg/mL of lac color. For subcutaneous administration of positive control group, OVA was first prepared at two-fold the target dose with 0.9% normal saline. Then, shortly before administration, the stock was emulsified with the same volume of FCA, resulting in a final concentration of 5 mg/mL of OVA. For intravenous administration of lac color group and lac color + FCA group, 250 mg of lac color powder was completely dissolved with 1 ml of DMSO, then they were diluted with 0.9% normal saline up to target concentration (1 mg/mL) and gently mixed just prior to administration. For intravenous administration of positive group, OVA was completely dissolved with 0.9% normal saline, then they were diluted with DMSO up to target concentration of 1 mg/mL OVA and 1.6% DMSO and gently mixed just prior to administration. For detailed protocol of treatment, see Active Systemic Anaphylaxis (ASA) test section below.

### Animals

For ASA test, male Hartley guinea pigs (weighing 250–300 g) were obtained from KOATECH (Gyeonggi-do, Korea. The animals were housed under controlled conditions (temperature, 16 ~ 26 °C; humidity, 30–70%) in the experimental animal facility at Seoul National University Hospital accredited by AAALAC International (#001169) in accordance with the Guide for the Care and Use of Laboratory Animals (8th ed.) [26]. These animals were allowed free access to their diet and tap water with a 12 h light:dark cycle. The animals were adapted to this environment for two weeks prior to study initiation. All animal studies were approved by the Institutional Animal Care and Use Committee of the Biomedical Research Institute at Seoul National University Hospital (Approval Number: 18–0177-S1A0) and conducted under Good Laboratory Practice (GLP) regulations for Nonclinical Laboratory Studies [27].

### Determination of dose ranges

Based on a comprehensive survey of food intake among the Korean population, we estimated the general consumption of lac color and determined the dosage for animal experiment. After examining the daily intake of specific food groups [28], we calculated the average daily lac color intake by applying the lac color content of those food groups [29], and the estimated human consumption of lac color was found to be 0.01 to 0.03 mg/kg/day. To administer a dose approximately 30 to 80 times higher in guinea pigs, we used the formula 4 × 8/37, which based on the body surface area calculation method as outlined by the FDA in "Estimating the Maximum Safe Starting Dose in Initial Clinical Trials for Therapeutics in Adult Healthy Volunteers" (2005) to translate the dose from animals to humans. According to this, guinea pigs were given a dose of 4 mg/kg, which is equivalent to a dose of 0.86 mg/kg in humans.

### Active systemic anaphylaxis (ASA) test

**Fig. 1:**
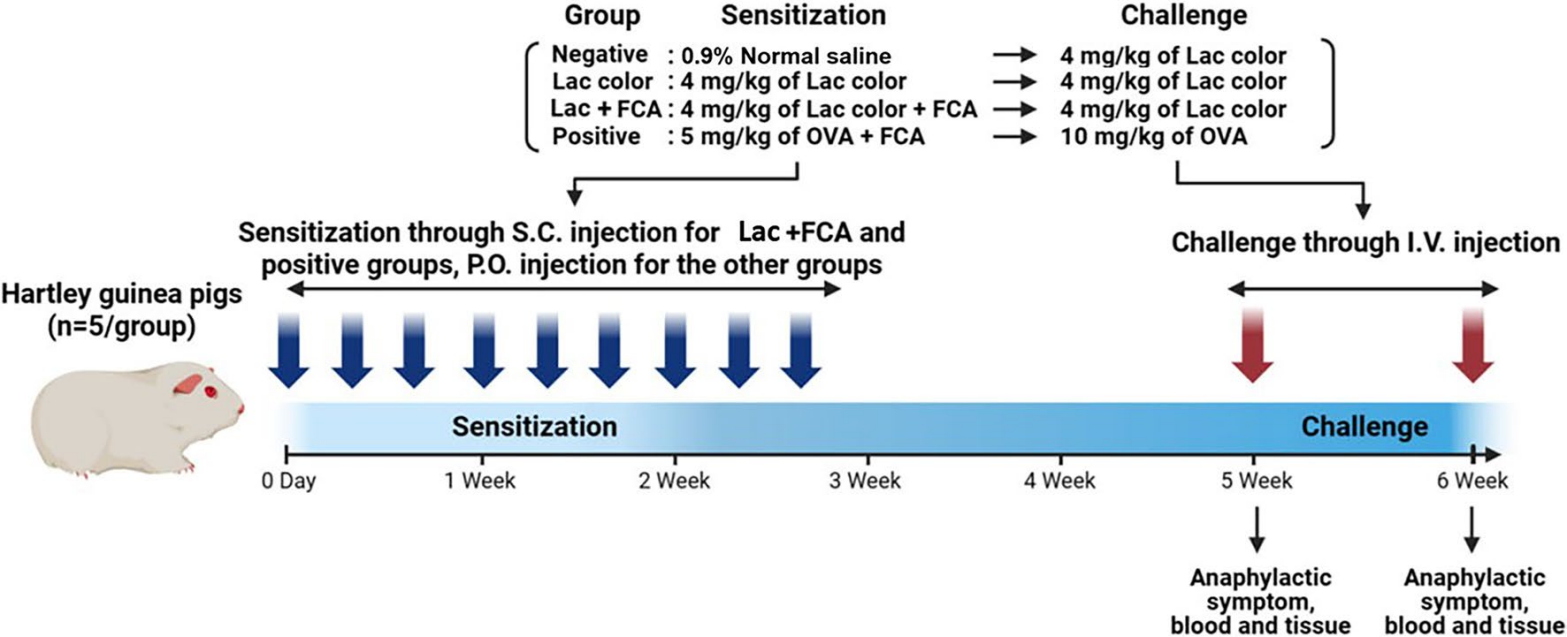
Scheme of active systemic anaphylaxis study of lac color. Two groups (negative control (0.9% normal saline), and Lac color (4 mg/kg)) were sensitized orally with lac color three times a week for three weeks. The Lac color + FCA dose group (4 mg/kg) and positive control group (5 mg/kg) were subcutaneously sensitized at a shaved back site with lac color plus FCA, or with OVA plus FCA respectively, once a week for three weeks. Fourteen days after the last sensitization, all test groups were challenged intravenously (IV) once a week for two consecutive weeks with lac color (for the negative control and lac color groups), and with OVA (for positive control group) to induce anaphylaxis

Guinea pigs (n = 5 per group) were allocated to the following groups. Two groups, including negative control group and lac color (4 mg/kg) group, were sensitized orally with 0.9% normal saline and lac color respectively three times a week for three weeks. The lac color + FCA group (4 mg/kg) and positive control group (5 mg/kg) were subcutaneously sensitized at a shaved back site with lac color + FCA, or with OVA + FCA respectively, once a week for three weeks. Fourteen days after the last sensitization, all test groups were challenged intravenously (IV) once a week for two consecutive weeks with lac color (for the negative control group, lac color group, and lac color + FCA group), and with OVA (for positive control group) to induce anaphylaxis. Animals that died following the first challenge were excluded from the second challenge. Intravenous injection for challenge was performed using manual restraint without anesthesia, via the auricular vein of the animals. The experimental design of the ASA test is summarized in Table 2A and Fig. 1.

**Table 2:** Experimental design of active systemic anaphylaxis test and anaphylactic symptom score criteria

Group	Sensitization				Challenge			
	Test substance	Dosage mg/mL/kg)	No. of animals	Route	Test substance	Dosage mg/mL/kg)	No. of animals	Route
*(A)*								
Negative control	0.9% normal saline	0	5	P.O.	Lac in 1.6% DMSO	4	5	I.V.
Lac color	Lac in 0.9% normal saline	4	5	P.O.	Lac in 1.6% DMSO	4	5	I.V.
Lac color + FCA	Lac + FCA in 0.9% normal saline	4	5	S.C.	Lac in 1.6% DMSO	4	5	I.V.
Positive control	OVA + FCA in 0.9% normal saline	5	5	S.C.	OVA in 1.6% DMSO	10	5	I.V.

Score criteria	Anaphylactic symptom
*(B)*	
Asymptomatic [−]	None
Mild [±]	Restlessness
	Piloerection
	Tremor
	Rubbing or licking nose
Moderate [+]	Sneezing
	Coughing
	Hyperpnea
	Urination
	Evacuation
	Lacrimation
Severe [+ +]	Dyspnea
	Rhonchus
	Cyanosis
	Staggering gait
	Jumping
	Gasping and writhing
	Convulsion
	Side position
	Cheyne-Stokes respiration
Death [+ + +]	Death

Lac, lac color; FCA, Freund’s complete adjuvant; OVA, ovalbumin; DMSO, dimethyl sulfoxide

Anaphylactic symptoms were observed for 30 min according to the Korea Food & Drug Administration, notice 2017–71 (2017. 8. 30.). Briefly, grading was determined based on the following four criteria: asymptomatic (−) is referred to as cases in which no symptoms were observed; mild (±) is referred to as cases in which restlessness, piloerection, tremor and rubbing or licking nose were observed; moderate (+) is referred to as cases in which sneezing, coughing, hyperpnea, urination, evacuation and lacrimation were observed; severe (+ +) is referred to as cases in which dyspnea, rhonchus, cyanosis, staggering gait, jumping, gasping and writhing, convulsion, side position and Cheyne-Stokes respiration were observed; and death (+ + +) (Table 2B).

### Exploratory study to identify potential biomarkers to supplement the ASA testing

To identify potential biomarkers of anaphylaxis, data were collected from the six groups of additional active systemic anaphylaxis tests comprising exploratory negative and exploratory positive groups (Table 3). The identified biomarkers were applied to the ASA test of lac colors and results were analyzed.

**Table 3:** Experimental design of exploratory study for identifying potential biomarkers of anaphylaxis

Study No.	Group	Sensitization				Challenge			
		Test substance	Dosage (mg/kg)	No. of animals	Route	Test substance	Dosage (mg/kg)	No. of animals	Route
1	Exploratory negative control	0.9% normal saline	0	5	P.O.	0.9% normal saline	0	5	I.V.
2	Exploratory negative control	0.9% normal saline	0	5	P.O.	Lac in 1.6% DMSO	4	5	I.V.
3	Exploratory negative control	0.9% normal saline	0	5	P.O.	Lac in 8% DMSO	20	5	I.V.
4	Exploratory positive control	OVA + FCA in 0.9% normal saline	5	5	S.C.	OVA	10	5	I.V.
5	Exploratory positive control	OVA + FCA in 0.9% normal saline	5	5	S.C.	OVA in 1.6% DMSO	10	5	I.V.
6	Exploratory positive control	OVA + FCA in 0.9% normal saline	5	5	S.C.	OVA in 8% DMSO	10	5	I.V.

Lac, lac color; FCA, Freund’s complete adjuvant; OVA, ovalbumin; DMSO, dimethyl sulfoxide

#### Hematological variables

Thirty minutes after the second challenge or following death, animals were anesthetized by isoflurane inhalation, blood was collected from interior vena cava using EDTA tube and hematological determinations were performed using an Animal Blood Counter (ADVIA2120i, Siemens Healthcare Diagnostics Ltd., Ireland). The following variables were analyzed: white blood cells (WBC), red blood cells (RBC), hemoglobin (HGB), hematocrit (HCT), platelets (PLT), mean corpuscular volume (MCV), mean corpuscular hemoglobin (MCH), neutrophils (NEU), eosinophils (EOS), basophils (BAS), lymphocytes (LYM), monocytes (MON), and reticulocytes (RETI).

#### Macroscopic variables

After exsanguination of animals, macroscopic lesions on the major organs in which anaphylaxis can develop, such as ear, gastrointestinal tract, spleen, lung, trachea, and heart were examined. The following variables were analyzed: presence/absence of redness, red spots, and distension.

#### Histopathological variables

Organs, including the ear, jejunum, spleen, lung and heart, were fixed in a 10% neutral buffered formalin solution and routinely embedded in paraffin. After sectioning into 4-μm thick slices, the slides were stained with hematoxylin and eosin. The following variables were examined by a qualified pathologist: edema, congestion, hemorrhage, and MICI in ear; sloughed villi, dilated lacteal, congestion, hemorrhage, eosinophilia, and MICI in the jejunum; congestion, hemorrhage, eosinophilia, and MICI in spleen; edema, congestion, hemorrhage, eosinophilia, and MICI in lung; congestion, hemorrhage, and hypereosinophilic myocytes in heart. The tissues were then graded according to lesion severity in a blinded fashion using the following criteria: Score 0: none, score 1: mild, score 2: moderate, score 3: severe.

#### Identification of potential biomarkers

The method to identify potential biomarkers utilized slight modifications of previous methods [30–32]. To find variables that exhibited differences between exploratory negative and exploratory positive control groups, statistical significance was determined by comparing the data from every respective exploratory negative group with the data from every respective exploratory positive group. Then, the variables that satisfied all statistical differences were identified as potential biomarkers.

#### Correlation analysis

Correlations between the identified biomarkers and anaphylactic symptom scores were analyzed. The anaphylactic symptom score was graded according to Table 2B, with the exception of rubbing or licking nose, urination and evacuation: Score 0: asymptomatic, Score 1: mild, Score 2: moderate, Score 3: severe, Score 4: death.

### Statistical analysis

Qualitative data in macroscopic variables were analyzed using chi-squared test and Fisher’s Exact test (expected frequency > 5 and < 5 respectively). Quantitative data in hematological analysis was analyzed using students t-test or one-way ANOVA (Dunnett’s for post hoc). Qualitative data in histopathological analyses were analyzed using Mann–Whitney U test or Kruskal–Wallis test (Mann–Whitney U with Bonferroni correction for post hoc). Correlation analyses were performed by Spearman’s rank correlation coefficient. *p* values < 0.05 were considered statistically significant. Statistical software SPSS version 26 (SPSS Inc., Chicago, IL, USA) was used to evaluate the statistical analysis.

## Results

### Active systemic anaphylaxis test with lac color

Fourteen days after the last sensitization, animals were intravenously challenged with test substances and anaphylactic symptoms were observed. In the negative control group, 2/5 animals showed rubbing or licking nose. In the lac color group, 3/5, 3/5, and 5/5 animals showed rubbing or licking nose, urination, and evacuation, respectively. In the lac color + FCA dose group, 2/5, 1/5, and 1/5 animals showed rubbing or licking nose, urination, and evacuation, respectively. In the positive control group, 5/5 animals died within six minutes (Table 4A). Seven days after the first challenge, the test substances were re-challenged intravenously, and anaphylactic symptoms were again observed. In the negative control group, 1/5, 2/5, and 2/5 animals showed rubbing or licking nose, urination, and evacuation respectively (Table 4B). In the lac color group, 2/5, 2/5, and 1/5 animals showed rubbing or licking nose, urination, and evacuation, respectively. In the lac color + FCA group, 4/5, 2/5, and 4/5 animals showed rubbing or licking nose, urination, and evacuation, respectively. All the animals in the positive control group died following the first challenge (Table 4A).

**Table 4:** Results of the active systemic anaphylaxis test with lac color

Group	Negative control	Lac color	Lac color + FCA	Positive control
Sensitizing substance	0.9% normal saline (0 mg/kg)	Lac (4 mg/kg)	Lac + FCA (4 mg/kg)	OVA + FCA (5 mg/kg)
Challenging substance	Lac (4 mg/kg)	Lac (4 mg/kg)	Lac (4 mg/kg)	OVA (10 mg/kg)
No. of animals	5	5	5	5
*(A)*				
Mild ( ±)				
Restlessness	0	0	0	1
Piloerection	0	0	0	0
Tremor	0	0	0	0
Rubbing or licking nose	2	3	2	1
Moderate ( +)				
Sneezing	0	0	0	0
Coughing	0	0	0	0
Hyperpnea	0	0	0	2
Urination	0	3	1	4
Evacuation	0	5	1	4
Lacrimation	0	0	0	0
Severe (+ +)				
Dyspnea	0	0	0	5
Rhonchus	0	0	0	0
Cyanosis	0	0	0	4
Staggering gait	0	0	0	4
Jumping	0	0	0	0
Gasping and writhing	0	0	0	5
Convulsion	0	0	0	5
Side position	0	0	0	5
Cheyne-Stokes respiration	0	0	0	5
Death (+ + +)				
Death	0	0	0	5

Group	Negative	High	High + FCA	Positive
Sensitizing substance	0.9% normal saline (0 mg/kg)	Lac (4 mg/kg)	Lac + FCA (4 mg/kg)	OVA + FCA (5 mg/kg)
Challenging substance	Lac (4 mg/kg)	Lac (4 mg/kg)	Lac (4 mg/kg)	OVA (10 mg/kg)
No. of animals	5	5	5	0
*(B)*				
Mild ( ±)				
Restlessness	0	0	0	–
Piloerection	0	0	0	–
Tremor	0	0	0	–
Rubbing or licking nose	1	2	4	–
Moderate ( +)				
Sneezing	0	0	0	–
Coughing	0	0	0	–
Hyperpnea	0	0	0	–
Urination	2	2	2	–
Evacuation	2	1	4	–
Lacrimation	0	0	0	–
Severe (+ +)				
Dyspnea	0	0	0	–
Rhonchus	0	0	0	–
Cyanosis	0	0	0	–
Staggering gait	0	0	0	–
Jumping	0	0	0	–
Gasping and writhing	0	0	0	–
Convulsion	0	0	0	–
Side position	0	0	0	–
Cheyne-Stokes respiration	0	0	0	–
Death (+ + +)				
Death	0	0	0	–

In the first challenge after 14 days from the last sensitization, guinea pigs in the negative control groups showed urination and evacuation. In the all lac color group, guinea pigs showed rubbing or licking nose, urination and evacuation. In the positive control group, guinea pigs all died within 30 min (**A**). In the second challenge after 7 days from the first challenge, guinea pigs in the negative control and the all lac color groups showed rubbing or licking nose, urination and evacuation (**B**). Lac, lac color; FCA, freund’s Complete Adjuvant; OVA, ovalbumin

### Exploratory study to identify potential biomarkers

#### Hematological biomarkers: decreased PLT and increased BAS count

**Fig. 2:**
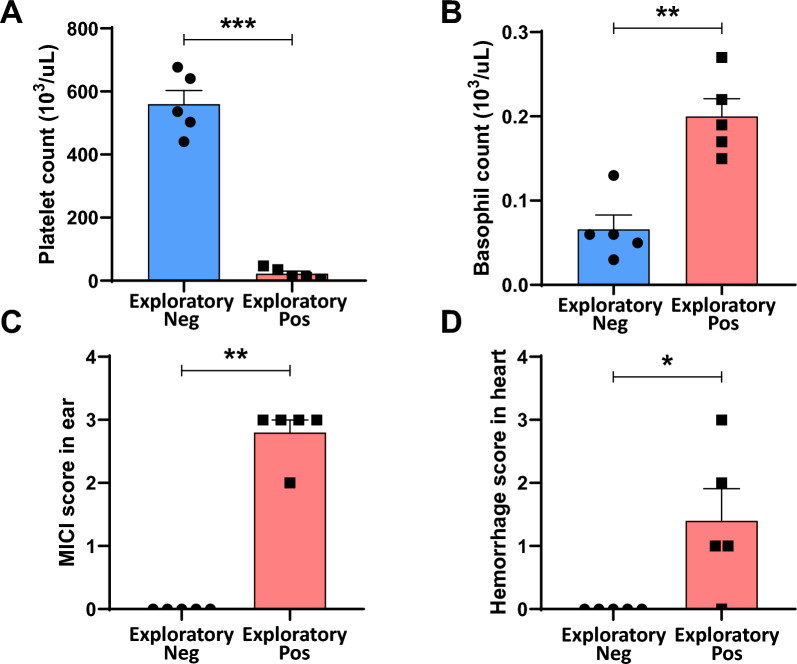
Identified potential biomarkers in hematology, histopathology. Platelet count (**A**), basophil count (**B**), MICI score in ear (**C**), hemorrhage score in heart (**D**) are identified as potential biomarkers due to statistically significant differences. (*p < 0.05, **p < 0.01, ***p < 0.001)

Variables such as platelet and basophils satisfied the requirement to show statistically significant differences (*p* < 0.05) for all comparisons and these two were strongly identified as potential biomarkers (Fig. 2, Supplementary Fig. 2). Statistical analysis results of the hematological data are shown in Supplementary Table 1.

#### Macroscopic biomarkers: distention in lung, and redness on the inner wall of trachea

**Fig. 3:**
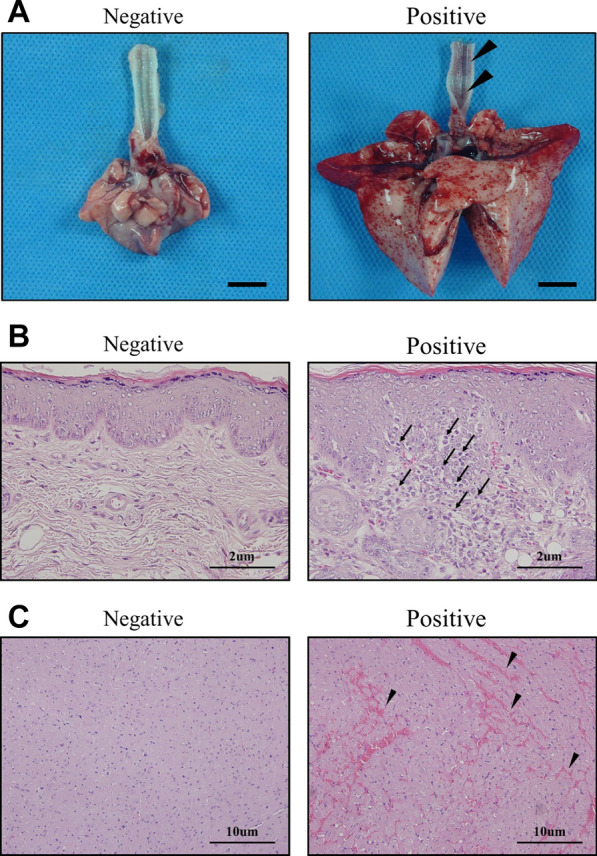
Representative macroscopic image of lung and histopathological images of ear and heart. **A** The images show that the lung and spleen became larger and redness on the inner wall of trachea occurred in the positive control group compared with the negative control group. **B** Microscopy of H&E stained ear sections show severe mononuclear inflammatory cell infiltration in positive control group (arrow). In contrast no lesions were observed in the negative control group (400×). **C** H&E stained heart sections show severe hemorrhage in positive control group (arrow heads). In contrast no lesions were observed in the negative control group (200×)

Variables such as distension in lung and redness on the inner wall of trachea satisfied the criteria of statistically significant differences (*p* < 0.05) for all comparisons and were thus identified as potential biomarkers (Table 5, Supplementary Table 2). The representative images of two macroscopic biomarkers can be seen in Fig. 3. Statistical analysis results of the macroscopic data are shown in Supplementary Table 3.

**Table 5:** Identified potential biomarkers in macroscopy

Group	Animal No	Distension in lung	Redness on the inner wall of trachea
		Incidence	Incidence
Exploratory negative control	110NMI001	Not Occured	Not Occured
	110NMI002	Not Occured	Not Occured
	110NMI003	Not Occured	Not Occured
	110NMI004	Not Occured	Not Occured
	110NMI005	Not Occured	Not Occured
Exploratory positive control	87PMI021	Occured	Occured
	87PMI022	Occured	Occured
	87PMI023	Occured	Occured
	87PMI024	Occured	Occured
	87PMI025	Occured	Occured
Statistical analysis results		Potential Biomarkers**	Potential Biomarkers**

**p < 0.01

#### Histopathologic biomarkers: MICI in lung and hemorrhage in heart

Two variables; MICI in ear and hemorrhage in heart, satisfied the criteria of all group comparisons exhibiting statistically significant differences (*p* < 0.05) for all comparisons and were thus identified as potential biomarkers (Fig. 2, Supplementary Fig. 2). Microscopy of H&E stained ear sections show that the ear and heart have severe mononuclear inflammatory cell infiltration and hemorrhage in the positive control group compared with the negative control group (Fig. 3). Statistical analysis results of the histopathological data are shown in Supplementary Table 4.

#### Correlations between anaphylactic symptom scores and the identified biomarkers

**Fig. 4:**
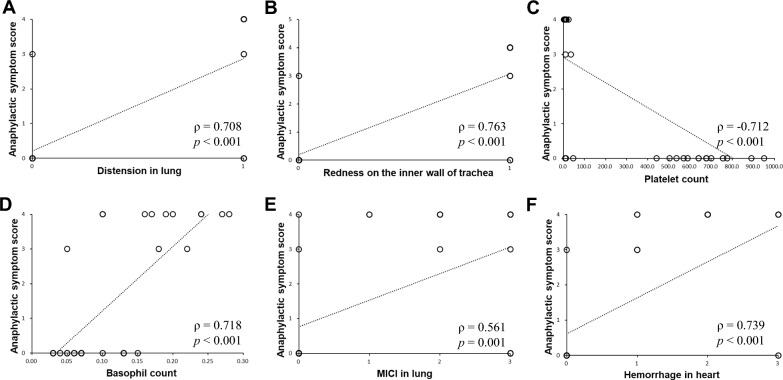
Correlations between anaphylactic symptom scores and identified biomarkers. Distension in lung (**A**), redness on the inner wall of trachea (**B**), Platelet count (**C**), basophil count (**D**), mononuclear inflammatory cell infiltration (**E**), and hemorrhage in heart (**F**) were confirmed as significantly correlated (*p* < 0.05). MICI: mononuclear inflammatory cell infiltration

Significant correlations were observed between the anaphylactic symptom scores and all of the identified biomarkers. Results for platelet count, basophil count, distention in lung, redness on the inner wall of trachea, and MICI in ear, hemorrhage in heart were ρ = -0.712, 0.718, 0.708, 0.763, 0.561, and 0.739 respectively; *p* < 0.05 for all cases (Fig. 4).

### Additional biomarker analysis in the ASA test of lac color

#### Hematological analysis

**Fig. 5:**
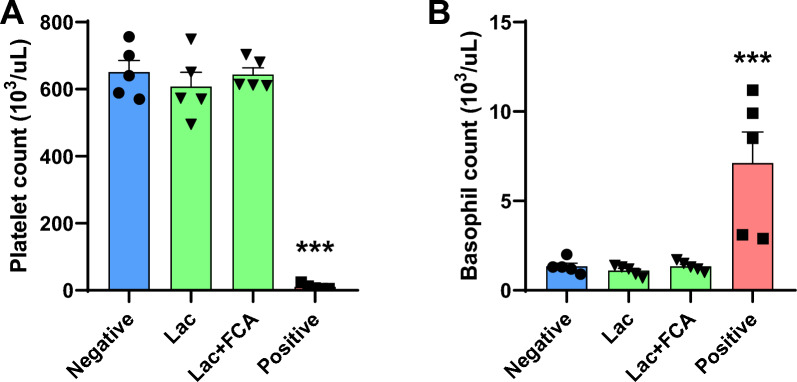
Hematologic analysis in the ASA test of lac color. In platelets (**A**) and basophil counts (**B**), the positive group demonstrated a statistically significant difference compared to the negative group. However, there were no statistically significant differences observed among all groups treated with lac color (***p < 0.001)

In platelet counts, none of the lac color groups exhibited significant differences compared to the negative control (651.0, 607.4, 643.8, for negative control, lac color, and lac color + FCA group respectively*; p* > 0.05 for all cases). Positive control was significantly increased compared to the negative control (651.0, 10.6 for negative control and positive control group respectively; *p* < 0.001) (Fig. 5A). In basophil count, none of the lac color groups demonstrated significant differences compared to the negative control (0.06, 0.04, 0.10, for negative control, lac color, and lac color + FCA group respectively; *p* > 0.05 for all cases). Positive control were significantly increased compared to negative controls (0.06, 0.19 for negative control and positive control group respectively; *p* < 0.001) (Fig. 5B).

#### Macroscopic analysis

None of the lac color groups exhibited distension in lung and significant differences compared to the negative control (1/5, 0/5, 0/5 for negative control, lac color, and lac color + FCA group respectively; *p* > 0.05 for all cases). In the positive control group, 5/5 animals showed distension in lung and a statistical difference was demonstrated upon comparison with the negative control group (*p* < 0.05) (Table 6). In negative control and all lac color groups, no animals showed redness on the inner wall of trachea and significant differences compared to the negative control (0/5, 0/5, 0/5 for negative control, lac color, and lac color + FCA group respectively; *p* > 0.05 for all cases). In positive control group, 5/5 animals showed redness on the inner wall of trachea and statistical difference was also observed when compared with the negative controls (*p* < 0.05) (Table 6).

**Table 6:** Macroscopic analysis in the ASA test of lac color

Group	No. of animals	Sensitizing substance	Challenging substance	Macroscopic biomarkers	
				Distension in lung	Redness on the inner wall of trachea
Negative	5	0.9% normal saline (0 mg/kg)	Lac (4 mg/kg)	1	0
Lac	5	Lac (4 mg/kg)	Lac (4 mg/kg)	0	0
Lac + FCA	5	Lac + FCA (4 mg/kg)	Lac (4 mg/kg)	0	0
Positive	5	OVA + FCA (5 mg/kg)	OVA (10 mg/kg)	5*	5*

Lac color groups showed no significant differences in lung distension or tracheal redness compared to negative controls. In contrast, positive control showed significant increases. Lac, lac color; OVA, ovalbumin

*p < 0.05

#### Histopathological analysis

**Fig. 6:**
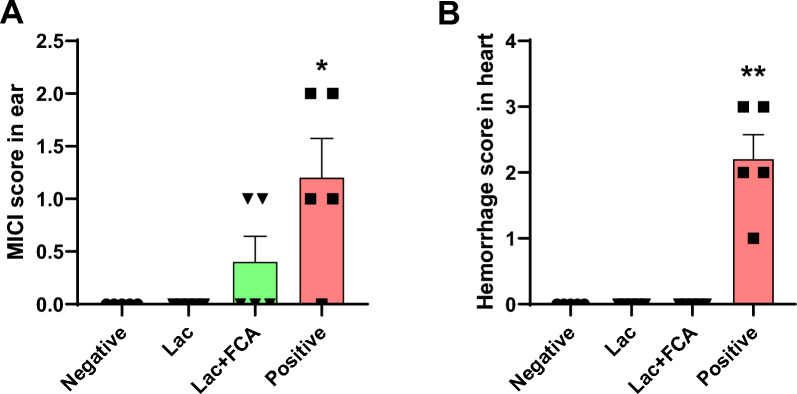
Histopathological analysis in the ASA test of lac color. MICI score in ear (**A**), and hemorrhage in heart (**B**), the positive group demonstrated a statistically significant difference compared to the negative group. However, there were no statistically significant differences observed among all groups treated with lac color (*p < 0.05, **p < 0.01)

For MICI in ear, none of the lac color groups have any significant differences compared to the negative control (0, 0, 0.4 for negative control, lac color, and lac color + FCA group respectively; p > 0.05 for all cases). Positive control group was significantly increased compared to the negative control group (0, 1.2 for negative control and positive control group respectively; *p* < 0.05) (Fig. 6A). For heart hemorrhage, none of the lac color groups have any significant differences compared to the negative control group (0, 0, 0 for negative control, lac color, and lac color + FCA group respectively; p > 0.05 for all cases). Positive control group was significantly increased compared to the negative control (0, 2.2 for negative control and positive control group respectively; *p* < 0.01) (Fig. 6B).

## Discussion

Anaphylaxis, a Type 1 hypersensitivity reaction, involves the release of IgE in response to an antigen, leading to mast cell and basophil degranulation. Mast cells, distributed throughout the body, play a crucial role in anaphylaxis, as do basophils circulating in the blood [33, 34]. When we evaluate anaphylaxis, MFDS recommend to use ASA or PCA test, which evaluate behavioral symptoms of the animals. The ASA test systematically activates mast cells and basophils, inducing a widespread allergic reaction. According to the MFDS guidelines [22], if just one case out of a group of five animals shows positivity, the judgment of the ASA test is considered positive. However, relying solely on behavioral tests is limited in terms of interpreting its anaphylactic possibilities. For example, from the results of the ASA testing we conducted, three symptoms (rubbing or licking nose, urination, and evacuation) were observed in lac color, and lac color + FCA group. No other symptoms were observed. However, these symptoms were also observed in the negative control group sensitized with 0.9% normal saline alone, preventing clear conclusions from the ASA test. Moreover, based on the experimental results from the second challenge, the frequency of rubbing or licking the nose, urination, and evacuation decreased in both the lac color and the lac color + FCA group. If lac color were an antigenic substance, these symptoms should have been observed more frequently upon the second challenge. Conversely, if lac color lacked antigenicity, these symptoms would have been expected to occur at similar frequencies as in the first challenge. This outcome suggests that rubbing or licking the nose, urination, and evacuation are not reliable indicators for evaluating anaphylaxis. Accordingly, to address this ambiguity, we conducted an exploratory study and further analyzed additional hematologic and pathologic values to investigate lac color’s possible immunogenic potential in spatially distributed organs, including blood, skin, heart, spleen, lungs, and trachea. These collectively provide a comprehensive understanding of systemic anaphylaxis. Based on the biological relevance of these values, we are further suggesting them as biomarkers for evaluating the immunogenicity of the test material.

Among the hematological data, we identified significant correlation between anaphylaxis and the number of basophils and a decreased platelet count. Basophils express high-affinity IgE receptor (FcεRI) and release allergic mediators upon interacting with antigens like mast cells [34]. In our analysis, an increased basophil count in blood was noted in the positive group, contrary to some previous anaphylactic studies which reported the decreased basophil number in anaphylactic group [35, 36]. This inconsistency might be due to the timing of blood collection time. In immunology, after secreting preformed mediators such as histamine that cause immediate allergic reactions (early phase), mast cells synthesize mediators such as TNF-α, LTB4, and IL-8. These mediators have chemotactic properties that recruit leukocytes such as basophils, monocytes, eosinophils, and lymphocytes into the tissues (late phase). This phase typically develops 2–6 h after antigen exposure and often peaks after 6–9 h [37]. In the present study, all blood samples were collected within 30 min of the challenge. Therefore, it can be assumed that the increased basophils measured in this study had not yet infiltrated the tissue due to the intravenous challenge. Further studies are needed to focus on the timing of basophil infiltration into tissues.

Additionally, a decreased platelet count was proposed as a potential biomarker. Previous studies have also demonstrated that the number of platelets in peripheral blood decreases in the event of anaphylaxis [38, 39]. The decrease in platelets is due to activated mast cells and basophils releasing platelet-activating factor, which aggregates platelets and reduces their count in the blood [38, 39]. The aggregated platelets are then activated and release mediators such as serotonin and platelet factor, which might promote the pathophysiology of anaphylaxis [21].

Lung distension, identified as a macroscopic biomarker in this study, was also observed in earlier work [33, 40]. During anaphylaxis, a significant volume of blood is withdrawn from systemic circulation, resulting in ischemia throughout the body and an increased demand for oxygen, which causes intercostal retractions. Additionally, histamine-induced bronchial contraction clogs the airways, increasing air pressure in the lungs and leading to lung distension [33, 41]. Similarly, redness on the inner wall of the trachea was identified as another macroscopic biomarker. Histamine induces vasodilation and increases vascular permeability, resulting in reddening [37]. These findings underscore the role of histamine in both lung distension and tracheal redness, highlighting its impact on vascular and bronchial changes during anaphylaxis.

Among histopathological biomarkers, mononuclear inflammatory cell infiltration (MICI) represented by lymphocytes, macrophages, and plasma cells were identified in previous anaphylaxis studies. According to Yang et al., mononuclear cellular infiltration was identified in the jejunal mucosa both at zero hours and peaking at 48, compared to a non-sensitized group in rats [42]. Wershil et al. reported neutrophil and mononuclear cell infiltration in the stomach of mice after antigen challenge, with neutrophils peaking at two hours and mononuclear cells rising several-fold at 12–24 h [43]. Like the infiltration of basophils into tissues, MICI is also a consequence of the late phase of allergies, which typically develops 2–6 h after allergen exposure, and often peaks after 6–9 h [37]. Therefore, it can be inferred that MICI in ear observed in this study is more affected by three weeks of sensitization than by I.V. challenge in that the tissue samples are from animals that died within 30 min. As another histopathological biomarker, hemorrhage in heart was identified. This is consistent with results identified in a previous report [44]. Mast cells are also located in the heart, perivascularly, in myocardial fibers, in the arterial intima, and in the adventitia [45]. This suggests that due to the influence of allergic mediators such as histamine, vascular permeability is increased, resulting in penetrating venous effluent to the heart tissue with resulting hemorrhage.

Based on the principle that anaphylaxis occurs systemically, the lesions in the various organs such as blood, skin, heart, lungs, and trachea were analyzed and biomarkers were identified. Furthermore, the identified biomarkers fully reflect anaphylaxis and therefore can be utilized to supplement the ASA test.

The biomarkers identified in this study are considered applicable to IgE-independent anaphylaxis as well. IgE-independent anaphylaxis occurs through the activation of Mas-related G protein-coupled receptor X2 (MRGPRX2) on the surface of mast cells and basophils by substances such as neuromuscular blocking drugs, opiates, antibiotics, iodinated contrast media, plasma expanders, and dyes. Additionally, large quantities of substances such as dextran, aprotinin, von Willebrand factor, and biologic therapeutics can activate Fcγ receptors on mast cells and basophils via IgG, leading to IgG-mediated anaphylaxis [46]. Despite these differences in the upstream pathways, both ultimately result in systemic anaphylaxis through the degranulation of mast cells and basophils and the release of histamine. Therefore, the biomarkers identified in this study are considered applicable to both types of anaphylaxis.

As a result of applying these biomarkers to the ASA test of lac color, none of the lac color groups including lac color, lac color + FCA showed no statistical difference from the negative control (Figs. 5, 6, and Table 6; *p* > 0.05 for all cases). On the other hand, the positive control, as expected, was significantly different. Therefore, when considering the results of the anaphylactic symptom score in the context of biomarker data, the ASA test is concluded to be negative. When all data are taken together, it is determined that lac color does not exhibit antigenicity at the concentrations we tested in the guinea pig model. These results are significant in that they represent the first study that has attempted to obtain safety data on the antigenicity of lac color, and they can also be utilized as a reference for government agencies to regulate the amount of lac color in consumer products.

## Conclusions

In summary, the results of the ASA test produced unclear results when monitoring symptoms assumed to be determinants of anaphylaxis such as rubbing or licking nose, urination and evacuation. Due to this, an exploratory study which aimed to identify potential biomarkers was conducted to supplement the ASA test. From the results, the following potential biomarkers were identified: decreased platelet count, and increased basophil count in hematology; distention in lung, and redness on the inner wall of trachea in macroscopy; mononuclear inflammatory cell infiltration (MICI) in ear and hemorrhage in heart in histopathology; all of which are immunologically highly associated with the anaphylaxis mechanism. Based on the use of these extensive biomarkers, none of the lac color groups showed any significant differences compared to the negative control group (*p* > 0.05). Taken together, it is determined that 4 mg/kg of lac color does not induce antigenicity in the guinea pig model. However, further research on the antigenicity of lac color in humans is encouraged. Furthermore, although the biomarkers identified and validated in this study may not be the only necessary and sufficient conditions for determining anaphylaxis, they can be utilized in future evaluations of antigenicity.

## Supplementary Information

Supplementary Information 1: Figure 1. Annual domestic sales change of lac color and carmine in Korea and Chemical structures of laccaic acids. **A** Annual domestic sales change of lac color and carmine in Korea [11, 12]. **B** Base structure of laccaic acid A, B, C, and E. **C** Structure of laccaic acid D.

Supplementary Information 2: Figure 2. Hematological and histopathologic data for biomarker identification in the exploratory study. **A** Platelet count in exploratory negative group II, III, exploratory positive group II, and III. **B** Basophil count in exploratory negative group II, III, exploratory positive group II, and III. **C** MICI score in ear in exploratory negative group II, III, exploratory positive group II, and III. **D** Hemorrhage score in heart in exploratory negative group II, III, exploratory positive group II, and III. (*p<0.05, **p<0.01, ***p<0.001).

## Data Availability

All data produced and analyzed in the current study are included in this paper. The data that support the findings of this study are available on request from the corresponding author on reasonable request.

## Abbreviations

ASA: Active systemic anaphylaxis
FCA: Freund’s complete adjuvant
GLP: Good laboratory practice
MICI: Mononuclear inflammatory cell infiltration
PCA: Passive cutaneous anaphylaxis
