# Supplementary material for: Antigenicity evaluation of lac color and exploratory study for identifying potential biomarkers of anaphylaxis
Source: Lab Anim Res. 2024 Nov 26;40:40. doi: 10.1186/s42826-024-00229-z (PMC11590302; doi:10.1186/s42826-024-00229-z)
Supplement: Supplementary file 1 — Supplementary Information 1: Figure 1. Annual domestic sales change of lac color and carmine in Korea and Chemical structures of laccaic acids. A Annual domestic sales change of lac color and carmine in Korea [11, 12]. B Base structure of laccaic acid A, B, C, and E. C Structure of laccaic acid D. [file 42826_2024_229_MOESM1_ESM.docx]

**Supplementary Table 1:** Hematological biomarker identification in the exploratory study

**(A)**

| Combination of comparison | WBC(10^3^/uL) |  | RBC(10^6^/uL) | | |  | HGB(g/dl) | | |  | HCT(%) |
| --- | --- | --- | --- | --- | --- | --- | --- | --- | --- | --- | --- |
|  | P value |  | P value | | |  | P value | | |  | P value |
| 1↔️6 | 0.628 |  | **0.013*** | | |  | **0.003**** | | |  | **0.003**** |
| 2↔️7 | **0.008**** |  | 0.649 | | |  | 0.227 | | |  | 0.077 |
| 3↔️8 | 0.270 |  | 0.454 | | |  | 0.541 | | |  | 0.265 |
| Results | ∙ | | |  | ∙ | | |  | ∙ |  | ∙ |
| Combination of comparison | PLT(10^3^/uL) | | |  | MCV(fl) | | |  | MCH(pg) | | |
|  | P value | | |  | P value | | |  | P value | | |
| 1↔️6 | **0.000***** | | |  | 0.059 | | |  | 0.603 | | |
| 2↔️7 | **0.000***** | | |  | **0.005**** | | |  | **0.012*** | | |
| 3↔️8 | **0.000***** | | |  | **0.005**** | | |  | **0.031*** | | |
| Results | **Potential**  **Biomarker** | | |  | ∙ | | |  | ∙ | ∙ |  |

**(B)**

| Combination of comparison | NEU(10^3^/uL) |  | EOS(10^3^/uL) |  | BAS(10^3^/uL) |
| --- | --- | --- | --- | --- | --- |
|  | P value |  | P value |  | P value |
| 1↔️6 | 0.266 |  | 0.453 |  | **0.004**** |
| 2↔️7 | **0.000***** |  | 0.150 |  | **0.025*** |
| 3↔️8 | **0.044*** |  | 0.156 |  | **0.019*** |
| Results | ∙ |  | ∙ |  | **Potential**  **Biomarker** |
| Combination of comparison | LYM(10^3^/uL) |  | MON(10^3^/uL) |  | RETI(10^9^/L) |
|  | P value |  | P value |  | P value |
| 1↔️6 | 0.081 |  | 0.225 |  | 0.132 |
| 2↔️7 | 0.986 |  | 0.158 |  | 0.304 |
| 3↔️8 | **0.029*** |  | 0.205 |  | 0.075 |
| Results | ∙ |  | ∙ |  | ∙ |

(A) Biomarker identification in complete blood cell count.

(B) Biomarker identification in white blood cell differential count and reticulocytes

Asterisks indicate a statistically significant difference (**p*<0.05, ** *p*<0.01, *** *p*<0.001). S.D., standard deviation; WBC, white blood cells; RBC, red blood cells; HGB, hemoglobin; HCT, hematocrit; PLT, platelet; MCV, mean corpuscular volume; MCH, mean corpuscular hemoglobin; NEU, neutrophils; EOS, eosinophils; BAS, basophils; LYM, lymphocytes; MON, monocytes; RETI, reticulocytes

**Supplementary Table 2.** Macroscopic data for biomarker identification in the exploratory study

**(A)**

| Group | Animal No. |  | Distension  in lung |  | Redness  on the inner  wall of trachea |  |
| --- | --- | --- | --- | --- | --- | --- |
|  |  |  | Incidence |  | Incidence |  |
| Exploratory Neg II |  |  | Not Occured |  | Not Occured |  |
|  |  |  | Not Occured |  | Not Occured |  |
|  |  |  | Occured |  | Not Occured |  |
|  |  |  | Not Occured |  | Not Occured |  |
|  |  |  | Not Occured |  | Not Occured |  |
| Exploratory Pos II |  |  | Occured |  | Occured |  |
|  |  |  | Occured |  | Occured |  |
|  |  |  | Occured |  | Occured |  |
|  |  |  | Occured |  | Occured |  |
|  |  |  | Occured |  | Occured |  |
| Statistical analysis results | |  | Potential  Biomarkers* |  | Potential  Biomarkers** |  |

*: *p*<0.05, **: *p<*0.01

**(B)**

| Group | Animal No. |  | Distension  in lung |  | Redness  on the inner  wall of trachea |  |
| --- | --- | --- | --- | --- | --- | --- |
|  |  |  | Incidence |  | Incidence |  |
| Exploratory Neg III |  |  | Not Occured |  | Not Occured |  |
|  |  |  | Not Occured |  | Not Occured |  |
|  |  |  | Not Occured |  | Not Occured |  |
|  |  |  | Not Occured |  | Not Occured |  |
|  |  |  | Not Occured |  | Not Occured |  |
| Exploratory Pos III |  |  | Occured |  | Occured |  |
|  |  |  | Occured |  | Occured |  |
|  |  |  | Occured |  | Occured |  |
|  |  |  | Occured |  | Occured |  |
|  |  |  | Occured |  | Occured |  |
| Statistical analysis results | |  | Potential  Biomarkers** |  | Potential  Biomarkers** |  |

**: *p*<0.01

**Supplementary Table 3:** Macroscopic biomarker identification in the exploratory study

| Comparisons | Redness  in lung |  | Red spots  in lung |  | Distension  in lung |  | Redness  on the inner  wall of trachea |  | Redness  in heart |
| --- | --- | --- | --- | --- | --- | --- | --- | --- | --- |
|  | P value |  | P value |  | P value |  | P value |  | P value |
| 1↔️6 | 1.000 |  | **0.048*** |  | **0.008**** |  | **0.008**** |  | N/A |
| 2↔️7 | 1.000 |  | **0.048*** |  | **0.048*** |  | **0.008**** |  | 0.167 |
| 3↔️8 | 0.444 |  | 0.167 |  | **0.008**** |  | **0.008**** |  | N/A |
| Results | ∙ |  | ∙ |  | **Potential**  **Biomarker** |  | **Potential**  **Biomarker** |  | ∙ |

Asterisks indicate a statistically significant difference (**p<*0.05, ***p<*0.01). S.D., standard deviation, N/A indicates uncalculated value because the data were a constant

**Supplementary Table 4:** Histopathological biomarker identification in the exploratory study

**(A)**

| Comparisons | Ear | | | | | | | |  |
| --- | --- | --- | --- | --- | --- | --- | --- | --- | --- |
|  | Edema |  | Congestion |  | Hemorrhage |  |  | MICI | |
|  | P value |  | P value |  | P value |  |  | P value | |
| 1↔️4 | 1.000 |  | **0.008**** |  | **0.032*** |  |  | **0.008**** | |
| 2↔️5 | 1.000 |  | 0.310 |  | 1.000 |  |  | **0.032*** | |
| 3↔️6 | 1.000 |  | **0.008**** |  | 0.690 |  |  | **0.008**** | |
| Results | ∙ |  | ∙ |  | ∙ |  |  | **Potential**  **Biomarker** | |

**(B)**

| Comparisons | Jejunum | | | | | | | | | | |
| --- | --- | --- | --- | --- | --- | --- | --- | --- | --- | --- | --- |
|  | Sloughed villi |  | Dilated lacteal |  | Congestion |  | Hemorrhage |  | Eosinophilia |  | MICI |
|  | P value |  | P value |  | P value |  | P value |  | P value |  | P value |
| 1↔️4 | 0.151 |  | 0.841 |  | **0.008**** |  | 1.000 |  | 1.000 |  | 1.000 |
| 2↔️5 | 0.310 |  | 0.310 |  | 1.000 |  | 1.000 |  | 1.000 |  | 1.000 |
| 3↔️6 | 0.548 |  | 0.841 |  | **0.016*** |  | 1.000 |  | 1.000 |  | 1.000 |
| Results | ∙ |  | ∙ |  | ∙ |  | ∙ |  | ∙ |  | ∙ |

**(C)**

| Comparisons | Spleen | | | | | | | |
| --- | --- | --- | --- | --- | --- | --- | --- | --- |
|  |  | Congestion |  | Hemorrhage |  | Eosinophilia |  | MICI |
|  |  | P value |  | P value |  | P value |  | P value |
| 1↔️4 |  | **0.016*** |  | 1.000 |  | 1.000 |  | 1.000 |
| 2↔️5 |  | 0.421 |  | 1.000 |  | 1.000 |  | 1.000 |
| 3↔️6 |  | 0.151 |  | 1.000 |  | 1.000 |  | 1.000 |
| Results |  | ∙ |  | ∙ |  | ∙ |  | ∙ |

**(D)**

| Comparisons | Lung | | | | | | | | | |
| --- | --- | --- | --- | --- | --- | --- | --- | --- | --- | --- |
|  |  | Edema |  | Congestion |  | Hemorrhage |  | MICI |  | Eosinophilia |
|  |  | P value |  | P value |  | P value |  | P value |  | P value |
| 1↔️4 |  | 1.000 |  | **0.008**** |  | **0.008**** |  | **0.008**** |  | 1.000 |
| 2↔️5 |  | 1.000 |  | **0.008**** |  | **0.032*** |  | 0.056 |  | 1.000 |
| 3↔️6 |  | 1.000 |  | 0.151 |  | 0.310 |  | 0.421 |  | 1.000 |
| Results |  | ∙ |  | ∙ |  | ∙ |  | ∙ |  | ∙ |

**(E)**

| Comparisons | Heart | | | | |
| --- | --- | --- | --- | --- | --- |
|  | Congestion |  | Hemorrhage |  | Hypereosinophilic myocytes |
|  | P value |  | P value |  | P value |
| 1↔️4 | 0.421 |  | **0.032*** |  | 0.056 |
| 2↔️5 | 0.690 |  | **0.008**** |  | **0.008**** |
| 3↔️6 | **0.032*** |  | **0.008**** |  | **0.008**** |
| Results | ∙ |  | **Potential**  **Biomarker** |  | ∙ |

**(A)** Biomarker identification in ear

**(B)** Biomarker identification in jejunum

**(C)** Biomarker identification in spleen

**(D)** Biomarker identification in lung

**(E)** Biomarker identification in heart

Asterisks indicate a statistically significant difference (**p*<0.05, ***p*<0.01, ****p*<0.001). S.D., standard deviation; MICI, mononuclear inflammatory cell infiltration
