# Supplementary figures and images for: Antigenicity evaluation of lac color and exploratory study for identifying potential biomarkers of anaphylaxis
Source: Lab Anim Res. 2024 Nov 26;40:40. doi: 10.1186/s42826-024-00229-z (PMC11590302; doi:10.1186/s42826-024-00229-z)

Supplementary Figure 1

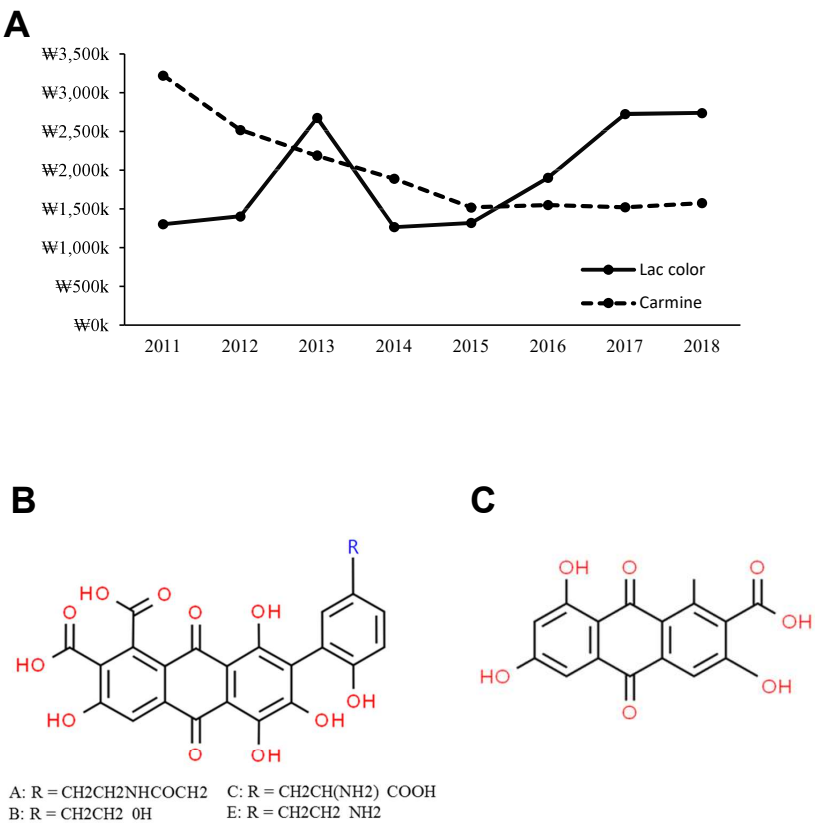

Supplementary Figure 2

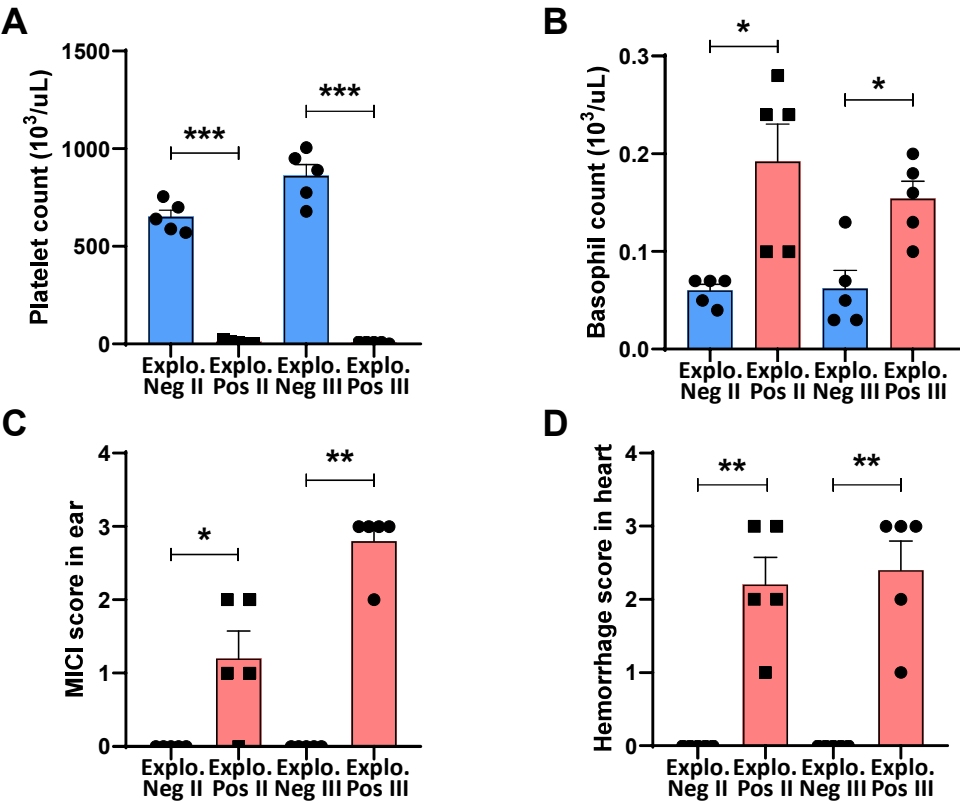

Supplement: Supplementary file 2 — Supplementary Information 2: Figure 2. Hematological and histopathologic data for biomarker identification in the exploratory study. A Platelet count in exploratory negative group II, III, exploratory positive group II, and III. B Basophil count in exploratory negative group II, III, exploratory positive group II, and III. C MICI score in ear in exploratory negative group II, III, exploratory positive group II, and III. D Hemorrhage score in heart in exploratory negative group II, III, exploratory positive group II, and III. (*p<0.05, **p<0.01, ***p<0.001). [file 42826_2024_229_MOESM2_ESM.pdf]
